# Hybridization altered the gut microbiota of pigs

**DOI:** 10.3389/fmicb.2023.1177947

**Published:** 2023-07-03

**Authors:** Limin Wei, Bo Zeng, Siyuan Zhang, Wei Guo, Feng Li, Jiangchao Zhao, Ying Li

**Affiliations:** ^1^Chongqing Key Laboratory of High Active Traditional Chinese Drug Delivery System, Chongqing Medical and Pharmaceutical College, Chongqing, China; ^2^Guangdong Provincial Key Laboratory of Animal Molecular Design and Precise Breeding, College of Life Science and Engineering, Foshan University, Foshan, China; ^3^College of Pharmacy, Chongqing Medical University, Chongqing, China; ^4^Farm Animal Genetic Resources Exploration and Innovation Key Laboratory of Sichuan Province, Sichuan Agricultural University, Chengdu, China; ^5^School of Laboratory Medicine/Sichuan Provincial Engineering Laboratory for Prevention and Control Technology of Veterinary Drug Residue in Animal-Origin Food, Chengdu Medical College, Chengdu, China; ^6^Key Laboratory of Southwest China Wildlife Resources Conservation (Ministry of Education), China West Normal University, Nanchong, China; ^7^Division of Agriculture, Department of Animal Science, University of Arkansas, Fayetteville, AR, United States

**Keywords:** gut microbiota, hybridization, pig, *16S rRNA*, metagenome, metabolome

## Abstract

Mammalian gut microbiota plays an important role in the host’s nutrient metabolism, growth, and immune regulation. Hybridization can enable a progeny to acquire superior traits of the parents, resulting in the hybridization advantage. However, studies on the effects of hybridization on the pigs’ gut microbiota are lacking. Therefore, this study used multi-omics technologies to compare and analyze the gut microbiota of the primary wild boar and its offspring. The *16S rRNA* gene sequencing results revealed that the gut microbiota of F4 exhibited a host-like dominance phenomenon with a significant increase in the abundance of *Lactobacillus* and *Bifidobacterium*. The beta diversity of Duroc was significantly different from those of F0, F2, and F4; after the host hybridization, the similarity of the beta diversity in the progeny decreased with the decrease in the similarity of the F0 lineage. The metagenomic sequencing results showed that the significantly enriched metabolic pathways in F4, such as environmental, circulatory system, fatty acid degradation adaptation, and fatty acid biosynthesis, were similar to those in F0. Moreover, it also exhibited similar significantly enriched metabolic pathways as those in Duroc, such as carbohydrate metabolism, starch and sucrose metabolism, starch-degrading CAZymes, lactose-degrading CAZymes, and various amino acid metabolism pathways. However, the alpha-amylase-related KOs, lipid metabolism, and galactose metabolism in F4 were significantly higher than those in Duroc and F0. Non-targeted metabolome technology analysis found that several metabolites, such as docosahexaenoic acid, arachidonic acid, and citric acid were significantly enriched in the F4 pigs as compared to those in F0. Based on Spearman correlation analysis, *Lactobacillus* and *Bifidobacterium* were significantly positively correlated with these metabolites. Finally, the combined metagenomic and metabolomic analysis suggested that the metabolic pathways, such as valine, leucine, and isoleucine biosynthesis and alanine aspartate and glutamate metabolism were significantly enriched in F4 pigs. In conclusion, the gut microbiota of F4 showed a similar host “dominance” phenomenon, which provided reference data for the genetics and evolution of microbiota and the theory of microbial-assisted breeding.

## 1. Introduction

Gut microbiota plays an important role in the symbiosis and co-evolution of the host as well as regulates the physiological functions of the host, such as nutrient metabolism, immune regulation, and disease resistance (Gilbert et al., 2018). The host genes can affect the composition and structure of gut microbiota *in vivo* (Goodrich et al., 2014; Davenport et al., 2015; Turpin et al., 2016; Lim et al., 2017). Zoetendal et al. studied the gut microbiota of twin and non-twin children, their parents, and completely unrelated individuals, and found the highest similarity in the composition of gut microbiota between the twins, while that between completely unrelated individuals was the lowest (Zoetendal et al., 2001; Turnbaugh et al., 2009). Bonder et al. studied the correlations between the genome and gut microbiota of the host and found that 1/3 of human gut microbiota could be inherited. They confirmed that the genes could interact with diet to regulate the abundance of *Bifidobacterium*, and multiple single-nucleotide polymorphisms (SNPs) were related to the abundance of *Rikenellaceae*, *Faecalibacterium*, *Lachnospira*, and *Eubacterium* (Bonder et al., 2016; Turpin et al., 2016). Kokou et al. (2018) observed “cold tolerance” in the gut microbiota of cold-tolerant *Tilapia* and showed that its gut microbiota had higher resilience to the changes in temperature, suggesting that the gut microbiota was shaped by a similar selection as the host.

Currently, hybridization is a commonly used breeding method in animal husbandry. In hybridization, the progeny can be allowed to inherit the superior traits of both parents by crossing, resulting in a hybrid advantage. Wild boar, the ancestor of domestic pigs, has stronger disease resistance and environmental adaptability, while domestic pigs have higher digestibility, faster growth rate, and shorter gestation period (Plogmann and Kruska, 1990; Li et al., 2013). The cross progeny of wild boars with domestic pigs, called hybrid wild boars, which can combine the strengths of wild boar and domestic pig, showing good dominant traits, with strong disease resistance, fast growth and development, high digestibility, and high production performance (Wu et al., 2011; Li et al., 2013).

However, no studies have been conducted on the effects of hybridization on the gut microbiota of pigs. In the current study, the gut microbiota of the primary wild boar and its progeny was compared and analyzed using multi-omics technologies. This study was conducted to further explore whether the gut microbiota also would undergo changes similar to the hybridization of the host in order to better understand the impact of hybridization on the gut microbiota of pigs *in vivo*.

## 2. Materials and methods

### 2.1. Ethics statement

All animal experiments were approved by the Institutional Animal Care and Use Committee of the Sichuan Agricultural University, Sichuan, China (DKY-2018102014).

### 2.2. Experimental objects and sample collection

**Figure 1:**
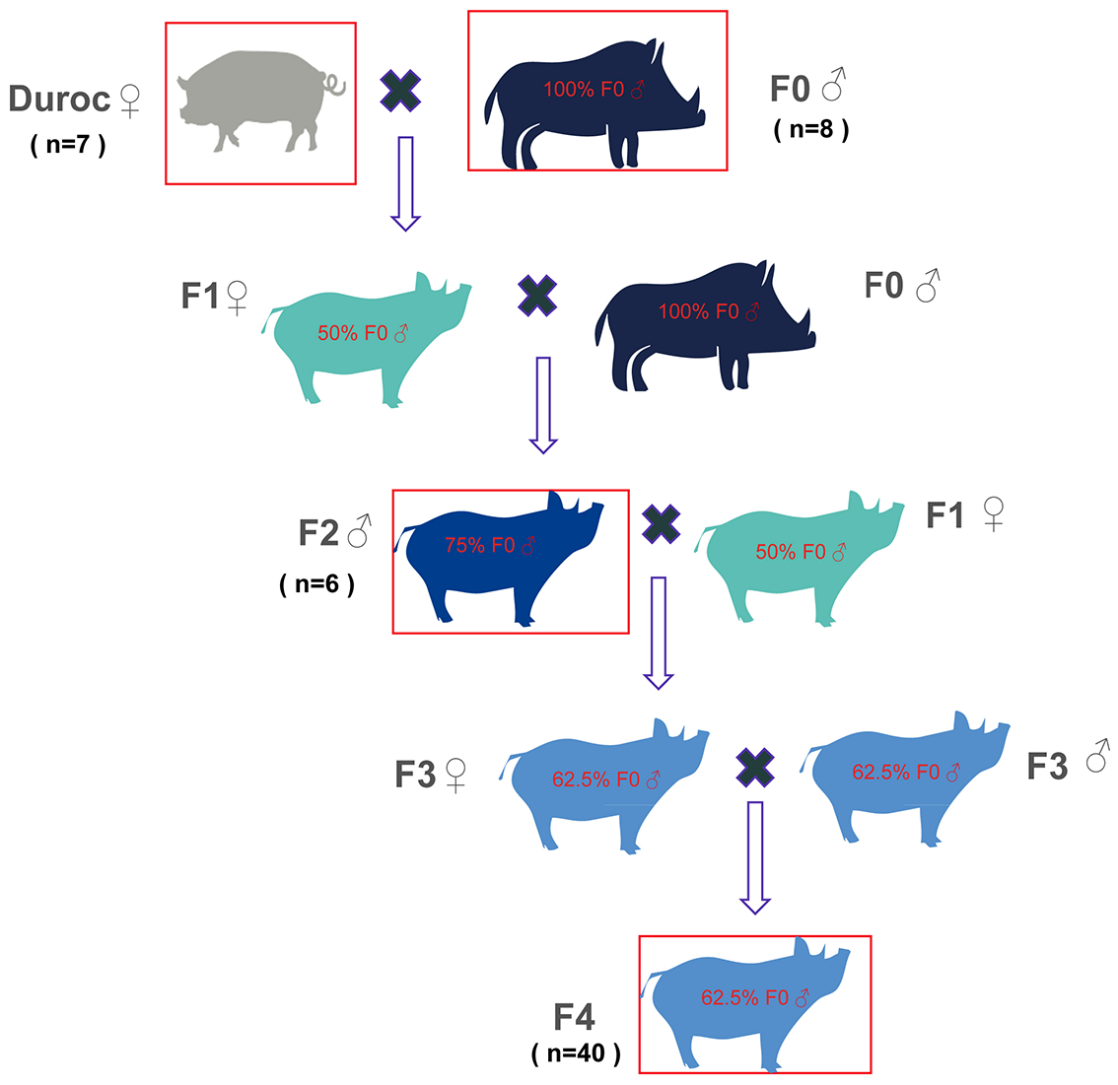
Schematic diagram of wild boar line hybridization. Original generation: wild boar (F0) was selected as the male parent, and domestic pig (Duroc) sow was selected as a female parent; Hybrid one generation wild boars (F1): F0 (♂) was crossed with Duroc (♀) to obtain F1 with 50% F0 contents. Hybrid second-generation wild boars (F2): F1(♀) were crossed with F0 (♂) to obtain F2 with 75% F0 contents. Hybrid third-generation wild boars (F3): F2 (♂) and F1(♀) were crossed to obtain F3 with 62.5% F0 contents. Hybrid fourth-generation wild boar feces (F4): F3 was self-crossed to obtain the F4, which contained 62.5% F0 contents. The red boxes denote those pigs, whose fecal samples were collected in this study and included Duroc, F0, F2, and F4.

In this study, the fourth-generation hybrid wild boar line with good and stable breeding performance was adopted from Sichuan Chengdu Jianyang Wild Boar Farm, as presented in Figure 1. Changbaishan purebred wild boar and American Duroc sow were selected as male and female parents, respectively. The fourth-generation hybrid wild boar (F4) with a pedigree content of 62.5% was then obtained using several methods, such as hybridization, backcrossing, cross-crossing, directional breeding, purification, rejuvenation, etc. The F4 generation possessed good hybrid advantages, such as stronger fecundity, annual output of more than 2.2 fetuses, faster growth rate, 8 months of commercial finishing pig weighing up to 90–100 kg, easier feeding, stronger disease resistance, fresher and tenderer meat, more types of amino acids, and more comprehensive nutrition, especially linoleic acid content, reaching 17.5%.

In the current study, the sixth generation of wild boars (F0), captured and artificially bred in the wild of Changbai Mountain in 2010, was collected. F0, Duroc, hybrid second-generation wild boar (F2), and F4 were divided into groups of six to eight pigs per pen, and all the pens were given the same diet (Supplementary Table S1) and kept in the same environment. Eight 4-year-old F0, seven 2-year-old Duroc, six 2-year-old F2, and 40 2-year-old F4 fecal samples were randomly collected (Supplementary Table S2). All animals were healthy and free of antibiotics. The fresh fecal samples were taken from the animal’s anus into a 40 mL sterile feces collection box and then and immediately placed on dry ice before being sent to Novogene Bioinformatics Technology, Co., Ltd. (Beijing, China) for *16S rRNA* gene sequencing, metagenomic sequencing, and metabolomic analyses.

### 2.3. Total genomic DNA extraction and sequencing of hypervariable V3–V4 region of bacterial *16S rRNA*

The total microbial genomic DNA was extracted from the fecal samples of Duroc (*n* = 7), F0 (*n* = 8), F2 (*n* = 6), and F4 (*n* = 40) using TIANamp Stool DNA Kit (TIANGEN Biotech, Beijing, China) and then sent to Novogene Bioinformatics Technology, Co., Ltd. (Beijing, China) for the sequencing of V3-V4 region of bacterial *16S rRNA* gene using the Illumina high-throughput platform (HiSeq2500). The V3–V4 regions of the *16S rRNA* gene were amplified using polymerase chain reaction (PCR) with primers 341F: CCTAYGGGRBGCASCAG and 806R: GGACTACNNGGGTATCTAAT, FastStart high-fidelity enzyme mix (New England Biolabs), and Phusion^®^ High-Fidelity PCR Master Mix (New England Biolabs). The PCR reaction conditions were as follows: initial denaturation at 98°C for 30 s, followed by 30 cycles of denaturation at 94°C for 45 s, annealing at 50°C for 60 s, and extension at 72°C for 90 s.

### 2.4. Analysis of bacterial *16S rRNA* gene sequencing data

The raw data of the *16S rRNA* gene sequencing were processed and analyzed using the open software Quantitative Insights Into Microbial Ecology 2 (QIIME2, 2021.11; Bolyen et al., 2019). Initially, noisy sequences, erroneous sequences, chimeric sequences, and repetitive redundant sequences were filtered out from raw data using DADA2 (Callahan et al., 2016). Moreover, OTUs were selected *de novo* using a similarity threshold of 97%. The SILVA database (silva_132_release) was used to annotate the classification of OTUs. Finally, based on the Shannon index and observed OTUs, bacterial alpha diversity was evaluated, and beta diversity was analyzed based on Jaccard, Bray Curtis, Weighted-Unifrac, and Unweighted-Unifrac distance algorithms.

### 2.5. Metagenomic analysis

Seven, eight, and ten fecal samples from Duroc, F0, and F4, respectively, were selected for metagenomic analysis. The qualified DNA samples were sent to Novogene Bioinformatics Technology, Co., Ltd. (Beijing, China). Metagenomic sequencing was performed using the Illumina NovaSeq 6,000 with a data volume of at least 10 G per sample after passing the Qubit test.

Genomic sequences of the host, containing low-quality base reads and N-base numbers up to 5% reads, were removed from raw data; the filtered sequences were then aligned to the porcine genome reference sequence using the MOCAT2 software (Kultima et al., 2016). The sequencing reads were assembled into contigs using the megahit v1 2.2 software (Li et al., 2016). The software Prodigal v2.6.1 (Hyatt et al., 2010) was then used to predict open reading frames (ORFs) in the assembled data. A non-redundant reference gene set (Zou et al., 2019) was constructed using the CD-HIT software (Fu et al., 2012). The normalized reads obtained previously were aligned to the non-redundant gene set using Bowtie2, and the relative abundance of genes in each sample was calculated (Qin et al., 2012).

For the functional annotation, the nucleotide sequence of the non-redundant gene set was translated into an amino acid sequence set using Biopython and annotated using the Kyoto Encyclopedia of Genes and Genomes (KEGG) database (Kanehisa et al., 2016). The non-redundant gene set was then compared with the carbohydrate-active enzymes (CAZy) database using the software HMMscan pipeline (Yin et al., 2012), and the CAZy genes encoded by the best match were retained. The annotated set of antibiotic resistance genes (ARG; Jia et al., 2017) was obtained by aligning the non-redundant gene set with the CARD database using blastp in the DIAMOND software (Buchfink et al., 2015).

### 2.6. Non-targeted metabolome analysis

The same fecal samples from F0 and F4, which were used for metagenomic sequencing, were selected and sent to Beijing Novo for non-targeted liquid chromatograph-mass spectrometer (LC–MS) analysis. After pre-treatment, the samples were analyzed using the Agilent 1,290 Infinity LC ultra-high-pressure liquid chromatography (UHPLC; Agilent, Palo Alto, CA, United States) and 6,600 Triple TOF mass spectrometer (AB Sciex, United States). Initially, the experimental and quality control (QC) samples were injected into the chromatographic column at a flow rate of 0.3 mL/min and a constant temperature of 4°C. The eluents consisted of A and B, where A contained water, ammonium acetate, and ammonia, and B was acetonitrile. The solvent gradient scheme was as follows: (1) solvent B was 85% for 1 min; (2) solvent B decreased from 85 to 65% in 0.1 min linearly; (3) solvent B decreased from 65 to 40% in 3 min linearly; (4) solvent B increased from 40 to 85% in 3 min linearly; and (5) solvent B remained constant at 85% for 5 min. Finally, the chemicals obtained previously were analyzed using mass spectrometry.

### 2.7. Processing of LC–MS data

The information on metabolites was obtained by identifying the matching peaks using several databases, such as mzCloud, mzVault, and MassList. The raw data were preprocessed for peak alignment, retention time correction, and peak area extraction using the XCMS program. The accurate molecular weights of the compounds were determined, and their molecular formulae were predicted. Metabolites were identified based on their fragment ions, collision energies, and other information. The compounds were then filtered using a coefficient of variant value < 30% as a threshold and used for subsequent normalization and quantitative analysis. The identified metabolites were functionally and taxonomically annotated. The identified metabolites were finally annotated using the KEGG database to analyze the functional properties and distribution of the metabolites.

### 2.8. Statistical analyses

The significance of alpha and beta diversity was detected using the Mann–Whitney test and analysis of community similarities (ANOSIM), respectively. Linear discriminant analysis (LDA) with effect size (LEfSe; LDA value > 2) was used to identify differences in the bacterial species, KEGG category, KEGG pathways, KEGG orthologs (KOs), CAZy enzymes, and ARG between the groups. The results were presented as a box plot, principal coordinate analysis map (PCoA), partial least squares discrimination analysis (PLS-DA; Boulesteix and Strimmer, 2007), heatmap, and histogram using R (3.6.0) software. The correlations among significantly differential metabolites, microbiota, and KOs were analyzed using the Spearman correlation analysis. The poor correlations among bacteria, metabolites, and genes were removed, while the strong pairwise correlations with *r* (relative coefficient) > 0.8 and *p* < 0.05 were selected. Profiles of network topology were visualized using Cytoscape (v3.6.1).

## 3. Results

### 3.1. Diversity analysis and composition classification of pig gut microbiota In each group of wild boar hybrid lines

The *16S rRNA* gene sequencing analysis from Duroc, F0, F2, and F4 resulted in a total of 4,325,796 high-quality reads with an average of 70,914 reads per sample, ranging from 45,049 to 88,000 OTUs. Clustering was performed on the sequences with 97% similarity, generating 45,159 OTUs (Supplementary Table S2).

**Figure 2:**
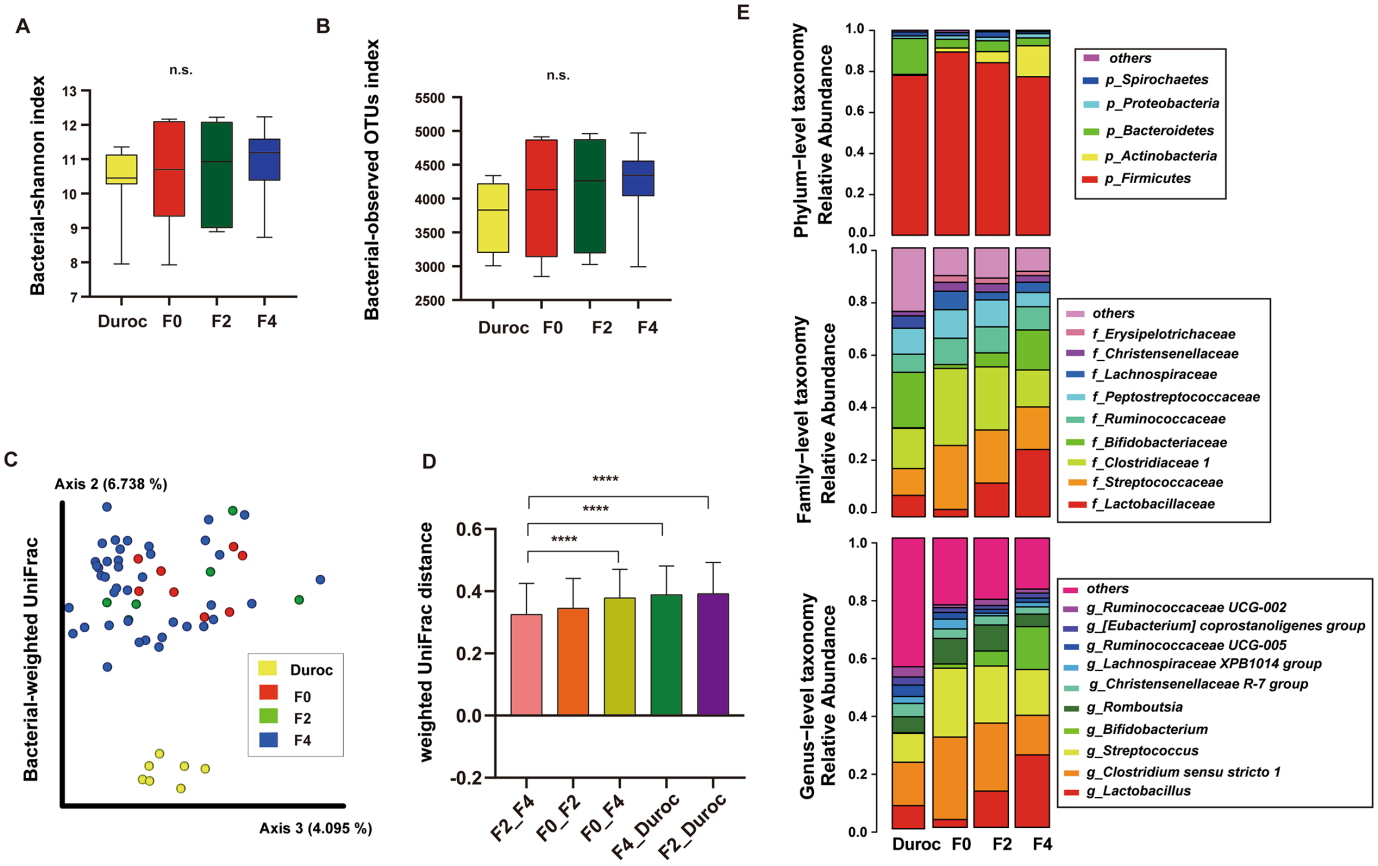
Diversity analysis and composition classification of pig gut microbiota in each group of wild boar hybrid lines. Alpha diversity included Shannon Index **(A)** and observed OTUs **(B)**. n.s., no significance, Mann–Whitney *U*-test. **(C)** PCoA of bacterial weighted UniFrac using ANOSIM, ^*^p < 0.05, ^**^p < 0.01, ^***^p < 0.001, ^****^p < 0.0001. The yellow, red, green, and blue circles represented the bacterial communities of the Duroc, F0, F2, and F4 pigs, respectively. **(D)** Comparison of weighted-Unifrac distances between groups, Mann–Whitney *U*-test, ^****^p < 0.0001. **(E)** Classification of the gut microbiota of wild boar hybrid lines using *16S rRNA* V3–V4 sequences at the phylum, family, and genus levels.

Alpha diversity was analyzed using Mann–Whitney *U*-test, which showed no significant differences in Shannon index and observed-OTUs among Duroc, F0, F2, and F4 groups (*p* < 0.05; Figures 2A,B; Supplementary Table S3).

Using the ANOSIM analysis of weighted UniFrac, beta diversity analysis showed that Duroc was significantly different from F0, F2, and F4, while in F0 and hybrid wild boar offsprings, only F0 and F4 were significantly different (*p* < 0.05), as shown in Figure 2C and Supplementary Table S3. The Bray Curtis, Jaccard, and unweighted UniFrac had the same results (Supplementary Figures S1A–C, ANOSIM; Supplementary Table S3).

The histogram of the weighted UniFrac distances between the groups showed that Duroc had the greatest relative distance to F2, and F4, whereas, the relative distance between the offspring decreased with the decrease in the relative content similarity of the F0 lineage. Moreover, F0 and F4 (containing 62.5% F0 contents) had the greatest distance, while F4 and F2 (containing 75% F0 contents) had the least distance (Figure 2D, Mann–Whitney *U*-test, *p* < 0.05; Supplementary Table S3).

Furthermore, all the animals were divided into male and female groups with a sample size of 33 and 28, respectively. Mann–Whitney *U*-test and ANOSIM analyses showed that the Shannon indices and Bray Curtis were not significantly different between the two groups (Supplementary Figures S1D,E; Supplementary Table S3). The results suggested that gender had no significant effect on their gut microbiota.

The distribution of OTUs at the phylum, family, and genus levels in the Duroc, F0, F2, and F4 groups are illustrated in Figure 2E. At the phylum level, Firmicutes had the highest abundance, accounting for about 82.2% of the total bacterial population. At the family level, three major families, such as *Lactobacillaceae*, *Streptococcaceae*, and *Clostridiaceae1* accounted for about 49.61% of the total bacterial population. At the genus level, *Lactobacillus*, *Clostridium sensu stricto 1*, and *Streptococcus* were the three most dominant genera, accounting for about 50.46% of the total bacterial population.

### 3.2. Changes in the high-abundance gut microbiota of pigs in each group of wild boar hybrid lines

It was found that during hybridization some of the high-abundance gut microbiota (relative abundance > 0.1%) had changed. Mann–Whitney *U*-test showed that in F4 pigs, the abundance of *Spirochaetes* reduced significantly after hybridization and was lower as compared to that in other groups, while the abundance of *Actinobacteria* increased gradually and was significantly higher than that in the other groups (*p* < 0.01; Supplementary Figures S2A,B).

**Figure 3:**
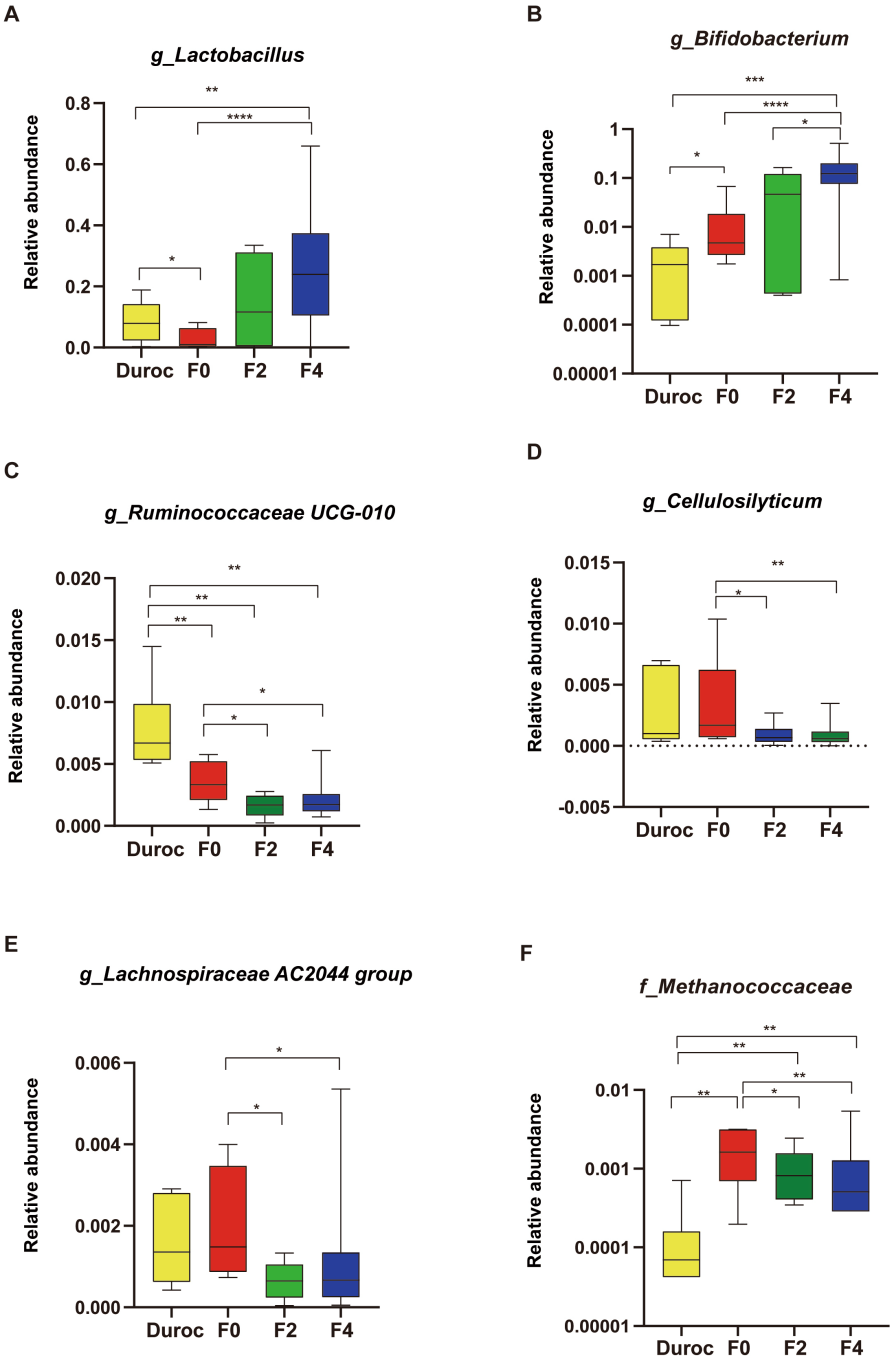
Changes in pig gut microbiota at the genus level. **(A)** *Lactobacillus*, **(B)** *Bifidobacterium*, **(C)** *Ruminococcaceae UCG-010*, **(D)** *Cellulosilyticum*, **(E)** *Lachnospiraceae AC2044 group*, and **(F)** *Methanococcaceae*. Mann–Whitney *U*-test, ^*^p < 0.05, ^**^p < 0.01, ^***^p < 0.001, ^****^p < 0.0001. The yellow, red, green, and blue bars represent Duroc, F0, F2, and F4, respectively. The line in the box represented the middle value, and the error bars represented the lowest and highest values, respectively.

At the genus level (Figure 3), similar to those of *Lactobacillaceae* and *Bifidobacteriaceae* (Supplementary Figures S3A,B), the relative abundances of *Bifidobacterium* and *Lactobacillus* gradually increased during hybridization and were the most abundant in the bodies of F4 pigs; their abundances were significantly higher in F4 pigs than those in Duroc and F0 (Whitney *U*-test, *p* < 0.01; Figures 3A,B). However, a gradually decreasing trend in the relative abundances of *Ruminococcaceae UCG-010*, *Cellulosilyticum*, and *Lachnospiraceae AC2044* was observed during hybridization, and their abundances were the lowest in F4 and significantly lower than F0 (Mann–Whitney *U*-test, *p* < 0.05; Figures 3C–E). Moreover, *Lachnospiraceae* and *Ruminococcaceae* showed a similar trend (Supplementary Figures S3C–E). *Methanococcaceae* had the highest abundance in F0, which was significantly higher than those in other groups; however, its abundance gradually decreased during the hybridization process, and the hybrid progeny was significantly higher than that in Duroc (Figure 3F).

### 3.3. Kyoto encyclopedia of genes and genomes function annotation of pig gut microbiota

A total of 2,342,775,516 paired raw reads were generated using Illumina NovaSeq 6,000 sequencing platform. A total of 2,136,113,996 clean reads were obtained after removing contaminated low-quality sequences. The high-quality sequences were then spliced, which generated 24,583,042 contigs, ranging between 454,042-1,318,590, and 49,520,094 ORFs, as listed in Supplementary Table S4.

Based on the composition analysis of the KEGG categories, the top 10 highly enriched KEGG pathways were obtained from the KEGG category (Supplementary Figure S4A). The results showed that the gut microbiota of F4 and Duroc pigs were significantly highly enriched in carbohydrate metabolism, nucleotide metabolism, and drug resistance antineoplastic as compared to those in F0. However, those of F4 and F0 were significantly highly enriched in environmental adaptation, circulatory system, transport, and catabolism as compared to those of Duroc. Moreover, the lipid metabolism in F4 was significantly more highly enriched than those in Duroc and F0 (Mann–Whitney *U*-test, *p* < 0.05; Supplementary Figures S4B–H).

**Figure 4:**
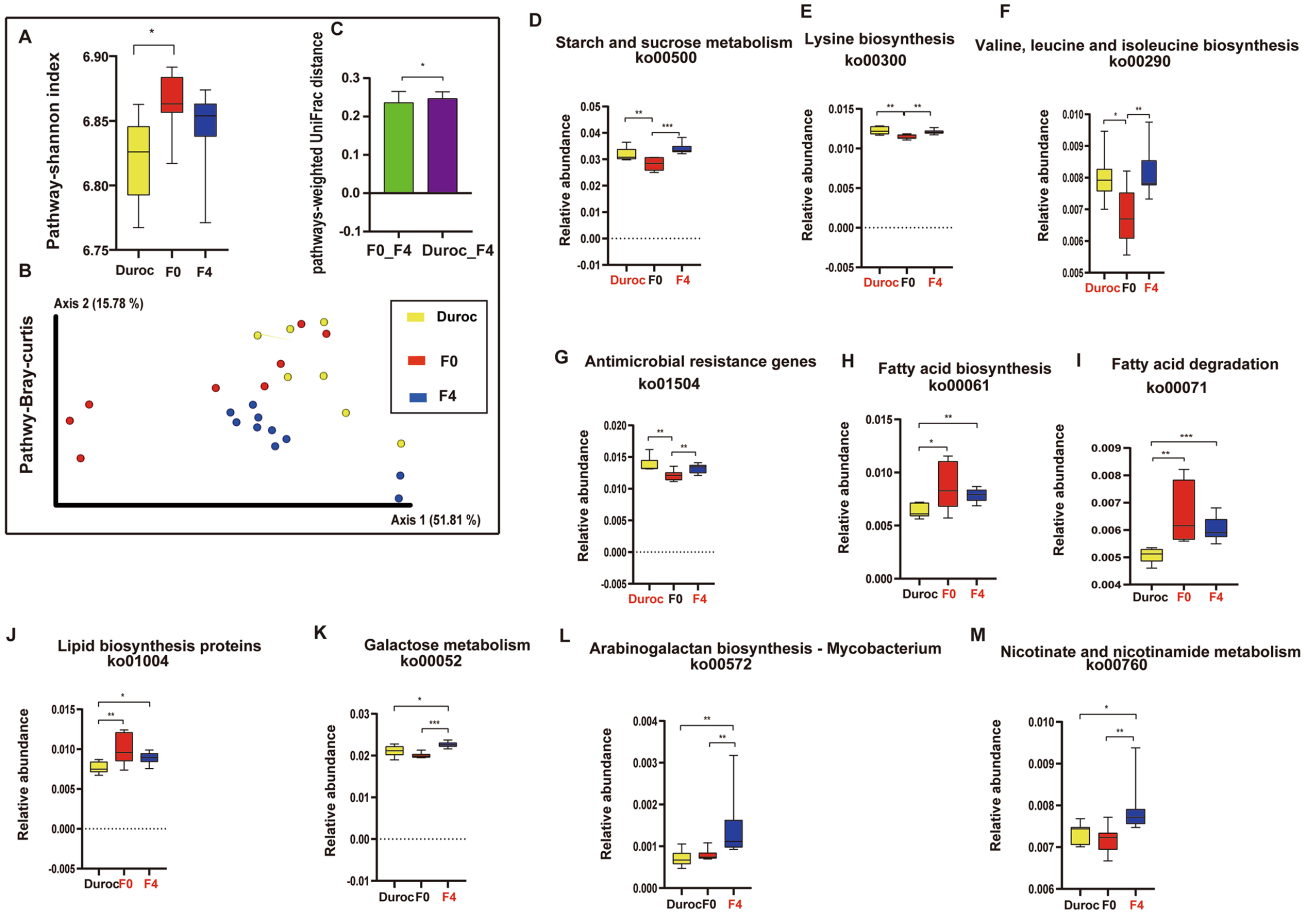
Diversity and variations in KEGG pathways. **(A)** Shannon index in alpha diversity. ^*^p < 0.05, Mann Whitney *U*-test. Yellow, red, and blue bars represent Duroc, F0, and F4, respectively. **(B)** PCoA of pathways based on Bray-Curtis distances. ANOSIM, ^*^p < 0.05, the yellow, red, green, and blue circles represent the bacterial communities of the Duroc, F0, F2, and F4 pigs, respectively. **(C)** pathway-weighted-Unifrac distances. **(D)** Starch and sucrose metabolism (ko00500). **(E)** Lysine biosynthesis (ko00300). **(F)** Valine, leucine, and isoleucine biosynthesis (ko00290). **(G)** Antimicrobial-resistant genes (ko01504). **(H)** Fatty acid degradation (ko00071). **(I)** Fatty acid biosynthesis (ko00061). **(J)** Lipid biosynthesis proteins (ko01004). **(K)** Galactose metabolism (ko00052). **(L)** Arabinogalactan biosynthesis *Mycobacterium* (ko00572). **(M)** Nicotinate and nicotinamide metabolism (ko00760). Mann-Whitney *U*-test, ^*^p < 0.05, ^**^p < 0.01, ^***^p < 0.001, ^****^p < 0.0001. The yellow, red, and blue bars represent Duroc, F0, and F4, respectively. Group names identified in red font represent significantly enriched groups. Boxplot indicates the interquartile range (IQR). The line in the box represents the middle value, and the error bars, respectively, represented the lowest and highest values.

Alpha diversity analysis showed that the Shannon index of the pathways in F0 increased significantly as compared to that of Duroc (Mann–Whitney *U*-test, *p* < 0.05; Figure 4A; Supplementary Table S5). The Bray-Curtis analysis of KEGG pathways revealed that Duroc, F0, and, F4 pigs were significantly different (ANOSIM, *p* < 0.05), as shown in Figure 4B and Supplementary Table S5. The histogram of the pathway-weighted UniFrac distances between the groups showed that the relative distance between the F4 and Duroc was greater than F4 and F0, suggesting that F4 and F0 have more similar functions (Figure 4C, Mann–Whitney *U*-test, *p* < 0.05; Supplementary Table S5).

After hybridization, some metabolic pathways, such as starch and sucrose metabolism, lysine, valine, leucine, and isoleucine biosynthesis, and ARGs, were significantly higher in F4 and Duroc as compared to those in F0 (Mann Whitney *U*-test, *p* < 0.05; Figures 4C–F). However, some pathways, such as fatty acid degradation, fatty acid biosynthesis, and lipid biosynthesis proteins, were significantly higher in F4 and F0 as compared to those in Duroc (Mann Whitney *U*-test, *p* < 0.05; Figures 4G–I). Moreover, some metabolic pathways in F4, such as galactose metabolism, arabinogalactan biosynthesis *Mycobacterium*, and nicotinate and nicotinamide metabolism, were significantly higher than those in Duroc and F0 (Mann Whitney *U*-test, *p* < 0.05; Figures 4J–L).

### 3.4. Carbohydrate-active enzymes and ARGs analysis of the pigs’ gut microbiota

**Figure 5:**
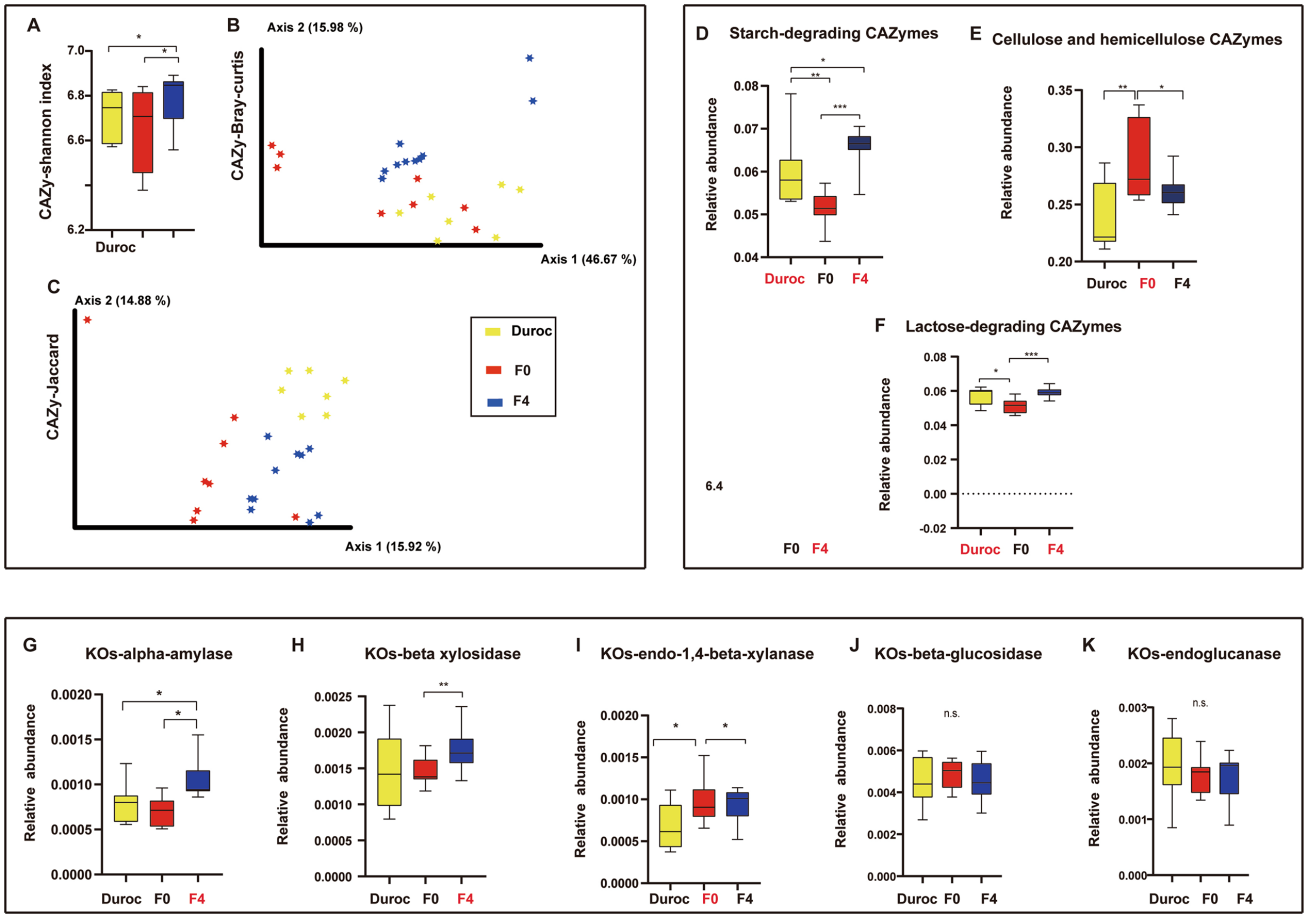
CAZymes diversity and changes in starch- and cellulose-hemicellulose-related CAZymes and KOs. **(A)** Alpha diversity is the Shannon index. ^*^p < 0.05, Mann Whitney U-test. Yellow, red, and blue bars represent Duroc, F0, and F4, respectively. **(B)** PCoA of CAZymes based on Bray-Curtis distances. **(C)** PCoA of CAZymes based on Jaccard distances. ANOSIM, *p* < 0.05. Yellow, red, and blue circles represent bacterial communities of pigs in the Duroc, F0, and F4 groups, respectively. **(D)** Starch-degrading CAZymes. **(E)** Cellulose and hemicellulose-GH CAZymes. **(F)** Lactose-degrading CAZymes. **(G)** KOs-alpha-amylase. **(H)** KOs-beta-xylosidase. **(I)** KOs-endo-1,4-beta-xylanase. **(J)** KOs-beta-glucosidase. **(K)** KOs-endoglucanase. Whitney *U*-test, ^*^p < 0.05, ^**^p < 0.01, ^***^p < 0.001, n.s., no significance. The yellow, red, and blue bars represent Duroc, F0, and F4, respectively. Group names identified in red font represent significantly enriched groups. Boxplot indicates the IQR. The line in the box represents the middle value, and the error bars, respectively, represent the lowest and highest values.

The Alpha diversity analysis showed that F4 had a significantly higher Shannon index of Carbohydrate-active enzymes (CAZymes) as compared to those of both Duroc and F0 (Figure 5A; Supplementary Table S6). The diversity of CAZymes was assessed between groups using the Bray–Curtis and Jaccard distance metrics and then visualized using PCoA. Significant differences were found between the CAZymes in Duroc, F0, and F4 (Figures 5B,C; Supplementary Table S6, ANOSIM, *p* < 0.05).

Moreover, the results showed that starch-degrading CAZymes were significantly more abundant in Duroc than those in F0. However, after crossing, F4 contained significantly more starch-degrading CAZymes, and both were significantly higher than those in Duroc and F0 as shown in Figure 5D (Mann Whitney *U*-test, *p* < 0.05); these results were similar to the alpha-amylase related KOs, as shown in Figure 5G. The analysis of cellulose and hemicellulose CAZymes revealed that the contents of cellulose and hemicellulose CAZymes in F0 were significantly higher than those in Duroc. After hybridization, the contents of cellulose and hemicellulose CAZymes increased in F4 and were higher than those in Duroc but significantly lower than those in F0 (Figure 5E; Mann–Whitney *U*-test, *p* < 0.05); this was consistent with the results of the endo-1,4-beta-xylanase related KOs (Figure 5I). Moreover, the lactose-degrading CAZymes analysis showed that the lactose-degrading CAZymes in Duroc and F4 were significantly higher than those in F0 (Figure 5F; Mann Whitney *U*-test, *p* < 0.05).

The Alpha diversity analysis showed that the Shannon index of ARG was significantly higher in both Duroc and F4 than that in F0 (Supplementary Figure S5A; Supplementary Table S7; Mann Whitney *U*-test; *p* < 0.05).The ARGs analysis indicated that ARGs in the Duroc, F0, and F4 showed significant differences in the Jaccard and Bray-Curtis diversity analyses (Supplementary Figures S5B,C; Supplementary Table S7, ANOSIM, *p* < 0.05). Tet (w/n/w), accounting for 31.6% on average, was the most abundant AGRS in Duroc, F0, and F4, followed by tet(40), accounting for 9.7% on average (Supplementary Figure S5D).

### 3.5. Partial least squares discrimination analysis and differential metabolites of wild boar and F4 group

**Figure 6:**
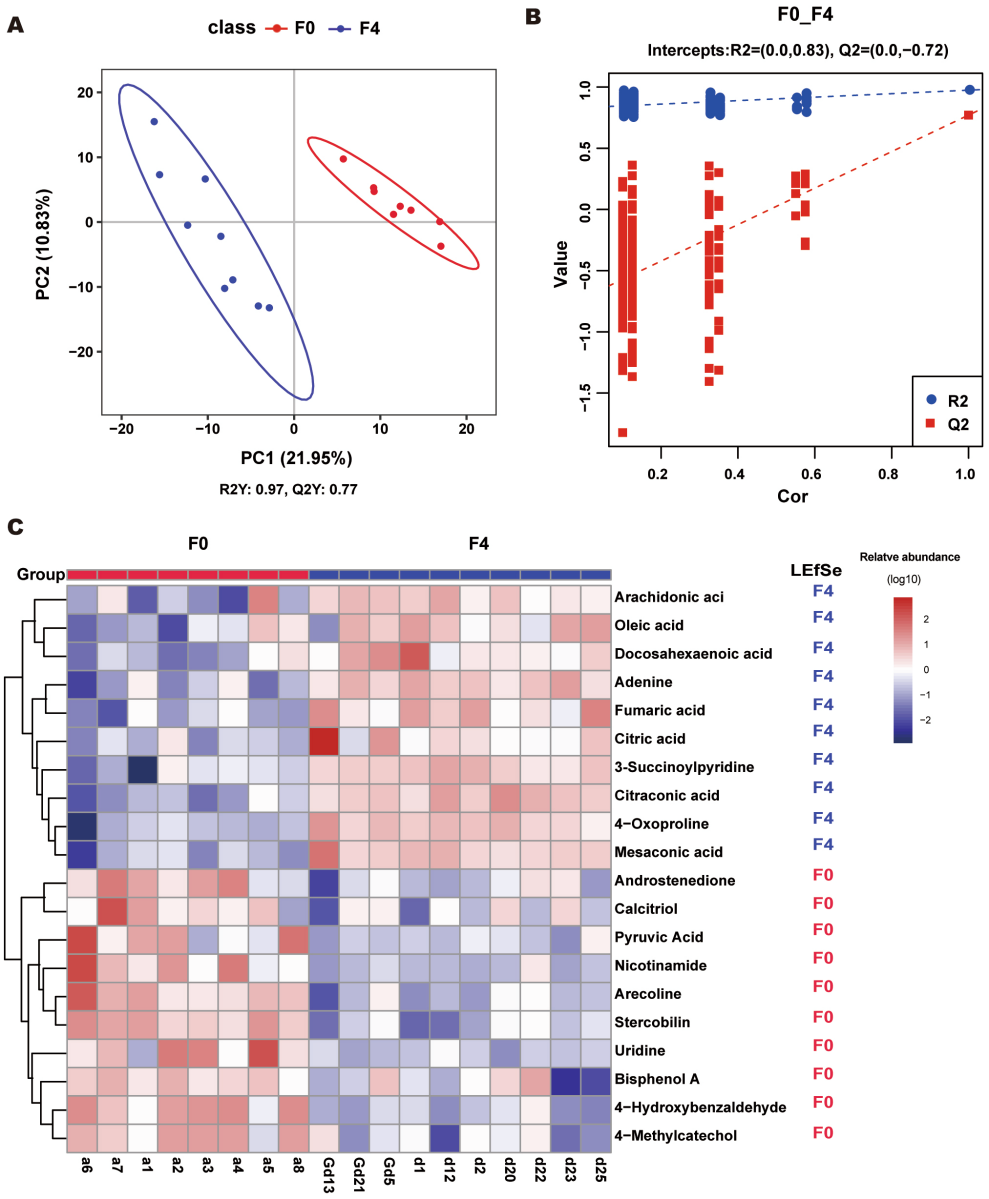
**(A, B)** PLS-DA score scatter plot and ranking validation plot. **(A)** To obtain the scatter plot; Abscissa is the sample’s score on the first principal component; the ordinate is the sample’s score on the second principal component; R2Y represents the interpretation rate of the model; Q2Y was used to evaluate the predictive ability of the PLS-DA model; and R2Y was greater than Q2Y. This indicated that the model was well-established. **(B)** For the ranking validation plot, in the ranking test, the abscissa represents the correlation of the randomized group y with the original group y, and the ordinate represents the scores of R2 and Q2. **(C)** Heatmap of top 20 different metabolites. The LEfSe column represents the group with a significant abundance of metabolite. “F4” and “F0” indicated that the abundance of this metabolite was significantly increased in F4 and F0, respectively (*p* < 0.05, LDA cutoff = 2.0).

As shown in Figure 6A, the metabolites of the F0 and F4 groups were divided into two clusters (ANOSIM, *p* < 0.05). The R2 > Q2 and Q2’s Y-axis coordinate < 0 showed that the model was not “overfit” and was a reliable model. The R2 was greater than Q2, and the coordinate of Q2 on the Y axis was less than 0 (Figure 6B); therefore, the model could be considered a reliable model and could better discriminate samples. Using the variable importance in the projection (VIP) value of the first principal component analysis in the PLS-DA model (VIP: the contribution rate of metabolite differences in different groups), the fold change (FC) was combined with the *p*-value of the LEfSe test to find differentially expressed metabolites. The threshold conditions were set as VIP > 1.0, FC > 2.0 or FC < 0.5, and *p*-value < 0.05. The obtained differential metabolites are listed in Supplementary Table S8.

The top 20 differential metabolites were plotted in a heatmap (Figure 6C). The contents of DHA, ARA, citric acid, oleic acid, fumaric acid, mesaconic acid, adenine, 4-oxoproline, citraconic acid, and 3-succinoylpyridine were significantly increased in the F4 pigs as compared to F0, while those of arecoline, 4-hydroxybenzaldehyde, nicotinamide, androstenedione, stercobilin, 4-methylcatechol, calcitriol, pyruvic acid, bisphenol A, and uridine decreased significantly.

### 3.6. Correlations among *16S rRNA gene*, metagenome, and metabolome

**Figure 7:**
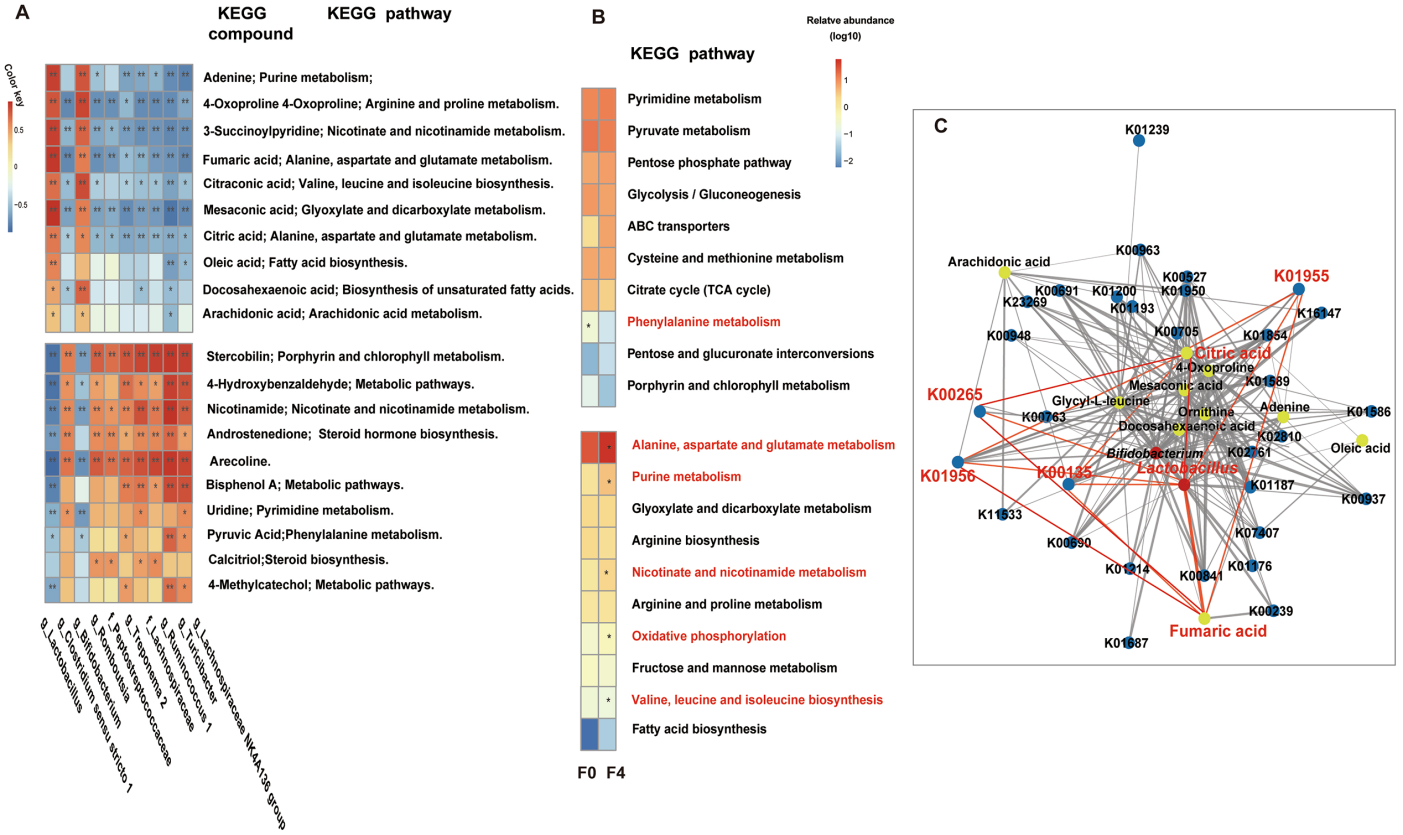
**(A)** Correlation analysis between genus-level bacteria and metabolites, and KEGG pathway enrichment. The red and blue fonts represent the metabolites and gut microbiota that were significantly enriched in the F0 and F4 groups. After FDR correction, Mann Whitney U-test, ^*^p < 0.05, ^**^p < 0.01, ^***^p < 0.001, ^****^p < 0.0001. **(B)** Metabonomic and metagenomic correlation analysis and *Lactobacillus*-related pathway metabolism. Spearman correlation analysis was performed on the relative content of the significantly differential KOs from the metagenome and significantly differential metabolites from the metabolome, and then the top 10 shared pathways between the significantly related KOs-attributed pathways and differential metabolites-attributed pathways were selected to draw a heatmap, as shown in **B**. The top half of the diagram shows the pathway shared by group F0, the corresponding classification, and LEfSe, and the bottom half shows the pathway shared by group F4, the corresponding classification, and LEfSe. The red font represents significantly enriched pathways in each group (*p* < 0.05, LDA cutoff = 2.0). **(C)** Co-occurrence networks within bacteria, metabolites, and genes. Red, yellow, and blue circles represent bacteria, metabolites, and genes, respectively. Nodes are connected by pairwise interactions (links). The weight of the link indicated a strong Pearson correlation (*r* > 0.8, *p* < 0.01), which was shared between bacteria, metabolites, and genes. The thickness of the link represented the strength of the correlation. Readers are suggested to read the web version of the article for interpretation of the high-resolution figures.

The top 10 genus-level differential gut microbes obtained using *16S rRNA* gene sequencing analysis were subjected to Spearman correlation analysis along with the top 20 differential metabolites with a one-to-one correspondence between samples. A positive correlation was observed between the significantly enriched gut microbiota and metabolites in each group (Figure 7A). The gut microbiota (*Clostridium sensu stricto 1*, *Romboutsia*, *Lachnospiraceae*, etc.), significantly enriched in F0, was positively correlated with metabolites, such as androstenedione, pyruvic acid, calcitriol, and nicotinamide, etc. These metabolites were mainly enriched in metabolic pathways, such as phenylalanine metabolism, steroid hormone biosynthesis, and vitamin digestion and absorption. However, the significant enrichment of *Lactobacillus* and *Bifidobacterium* in F4 was positively correlated with metabolites, such as DHA, ARA, oleic acid, fumaric acid, citric acid, etc. These metabolites were mainly enriched in the metabolic pathways, such as biosynthesis of unsaturated fatty acids, ARA metabolism, linoleic acid metabolism, and biosynthesis of fatty acids.

Spearman correlation analysis was performed on the relative contents of significantly differential KOs (Supplementary Table S9), significantly differential metabolites between the F0 and F4 groups, and the top 10 KEGG pathways. The significantly positively correlated KOs and metabolites were then plotted into a heatmap for further analysis (Figure 7B). A shared pathway, the phenylalanine metabolism pathway, was significantly enriched in the F0 group (*p* < 0.05, LDA cutoff = 2.0) as compared to other pathways. However, the F4 group was significantly enriched with five shared pathways, including alanine aspartate and glutamate metabolism, valine, leucine, and isoleucine biosynthesis, nicotinate and nicotinamide metabolism, purine metabolism, and oxidative phosphorylation.

The significantly enriched differential bacteria, metabolites, and genes of F4 were imported into the psych and reshape2 packages of R software for screening the data with *r* > 0.8 and *p* < 0.01. The network diagram of bacteria, metabolites, and genes was then made using Cytoscape software (Figure 7C). Spearman correlation analysis showed that in F4, the significantly enriched *Lactobacillus*, alanine aspartate, and glutamate metabolism as well as their associated KOs (K00135, K01956, K01955, and K00265), and metabolites, such as citric acid and fumaric acid, were significantly positively correlated.

## 4. Discussion

The composition and structure of gut microbiota are closely related to the host genes(Turpin et al., 2016). Studies revealed that gut microbiota had a higher similarity in closely related members as compared to the unrelated members (Ley et al., 2005; Turnbaugh et al., 2009; Raznahan et al., 2018). Similar results were obtained in the current study. The beta diversity of gut microbiota showed significant differences between Duroc and F0, F2, and F4; after the host hybridization, similarity in the beta diversity of the progeny decreased with the decrease in the similarity of the F0 lineage.

The gut microbiota co evolved with the host and was able to stably transmit to offspring with the host(A. Bjork et al., 2011; Langergraber et al., 2012). According to the report, in the experiment of inbred mice, it has been found that the progeny of inbred mice can trace back to the ancestral mice, and can be transmitted steadily to the 10th generation(Moeller et al., 2018). The current study and previous studies reported that domestic pigs were rich in *Lactobacillus*, while wild boars were rich in *Bifidobacterium* (Ushida et al., 2016; Chen et al., 2021; Wei et al., 2022). Interestingly, we found the dominant *Lactobacillus* and *Bifidobacterium* of the primary generation can be stably inherited to F4, and the abundance in F4 was significantly higher than that in the primary generation. *Bifidobacterium* and *Lactobacillus* are two probiotics that play an important role in improving animal growth performance and immune function (Li et al., 2015; Faseleh Jahromi et al., 2016; Feng et al., 2019; Abou-Kassem et al., 2021), which may be closely related to the Heterosis of F4. The gut microbiota of F4 showed a dominant phenomenon, implying that the gut microbiota might be shaped by similar selection as the host (Kokou et al., 2018). Wagner et al. studied the microbial composition of hybrid maize and reported a similar phenomenon. Moreover, the microbial composition of the hybrid progeny maize exhibited a dominance phenomenon consistent with the maize phenotype, which illustrated that the microbial composition might be affected by some traits of the host (Wagner et al., 2020).

A similar phenomenon was also found in the metabolic function of gut microbiota. The relative abundances of metabolic pathways, such as environmental adaptation, circulatory system, fatty acid degradation, and fatty acid biosynthesis in wild boar and F4 were significantly higher than those in Duroc, indicating that the gut microbiota function might also occur along with the selective evolution of host genetics. Studies showed that short-chain fatty acids (SCFA) could affect the motility of the gastrointestinal tract (GI; Cherbut et al., 1998), maintain the integrity of the intestinal barrier, reduce inflammation, and improve autoimmune diseases and allergies (Nicolas and Chang, 2019). This might play an important role in the disease resistance and immune function of F0 and F4. In F4 and Duroc, the significant enrichment of carbohydrate metabolism, starch and sucrose metabolism, starch-degrading CAZymes, and lactose-degrading CAZymes might be closely related to the digestibility of the body’s feed. Significant enrichment of some amino acid metabolic pathways in F4 and Duroc was also found. Among them, valine, leucine, and isoleucine are the essential oxyacids of the animal organism, which could promote the synthesis of proteins and utilization of dietary amino acids (Xu et al., 2019) and improve the production and reproduction performance of the animal (Park and Austic, 2000). A study also indicated that *Lactobacillus* could promote the synthesis of amino acids, such as valine, leucine, and isoleucine (Kumar Suryawanshi and Kango, 2021), which might be the reason for its significant enrichment in F4. Interestingly, the current study shows that galactose metabolism in F4 was significantly higher than that in F0 and Duroc; this might be also related to the significantly enriched *Lactobacillus* and *Bifidobacterium* in F4. Numerous studies reported that both *Lactobacillus* and *Bifidobacterium* could ferment lactose into galactose (de Vries and Stouthamer, 1968; Turner and Martley, 1983; Hickey et al., 1986), which is involved in several physiological functions, such as protecting the intestinal epithelial barrier and improving immune function (Salkovic-Petrisic, 2021; Szczykutowicz et al., 2021). Among them, Galacto-Oligo Saccharides (GOS) could also promote the proliferation of *Lactobacillus* and *Bifidobacterium* as well as the production of lactic acid and butyric acid (Dai et al., 2019). In addition, although antibiotics were not used, we found that the diversity of ARGs and “antimicrobial resistance genes” in the F4 and Duroc were higher than those in F0, which may be due to artificial captivity. Guo et al. also found a similar phenomenon in the wild and captive giant pandas (Guo et al., 2019).

The analysis of microbial metabolome showed that F4 also exhibited a dominant phenomenon. The F4 pigs were significantly enriched with DHA, ARA, citric acid, and fumaric acid. Spearman correlation analysis found that the probiotics *Lactobacillus* and *Bifidobacterium* in F4 were positively correlated with these metabolites, indicating that these two bacterial groups might contribute to the production of these metabolites. Studies demonstrated that increasing *Lactobacillus* and *Bifidobacterium* could significantly improve the contents of DHA and ARA in the body (Fukushima et al., 1999; Wall et al., 2010; Barrett et al., 2012; Ivanovic et al., 2015). Moreover, some other studies proved that *Lactobacillus* could also produce citric acid and fumaric acid (Yu et al., 2015; Yamamoto et al., 2021), which was consistent with the results of the current study. DHA and ARA belong to ω-3 and ω-6 polyunsaturated fatty acids, respectively, which are both essential unsaturated fatty acids in humans and animals. Studies revealed that the addition of DHA or ARA to the diet could promote the growth, development, and immune function of animals as well as improve their reproductive performance (Navas et al., 1997; Harel et al., 2001; Lee et al., 2019; Koletzko et al., 2020; Huang et al., 2021). The two kinds of organic acids, including citric acid and fumaric acid, have several good effects on animal diets, such as reducing intestinal pH, inhibiting the proliferation of harmful bacteria *Escherichia coli*, improving the growth rate and food intake, and enhancing immunity (Henry et al., 1985; das Neves et al., 2021; Fikry et al., 2021; Krauze et al., 2021).

Alanine aspartate and glutamate metabolism, which was the shared metabolic pathway of metabolome and metagenome correlation analysis, was significantly enriched in the F4 pigs. This pathway might have important functions related to digestive absorption, growth metabolism, and immune disease resistance of the host. Glutamate is an important amino acid in mammals with a strong antioxidant capacity (Windmueller and Spaeth, 1974). It could not only provide nutrients and energy for intestinal and mucosal cells (Wu, 1998), but also promote the proliferation of immune cells, affect the maturation of B cells (Newsholme, 2001), and improve the immune function of animals. Studies have shown that dietary glutamate supplementation could improve the growth performance, nutrient metabolism, and average daily gain of piglets while maintaining intestinal barrier integrity and reducing diarrhea and weaning syndrome (Junjie, 2019). Moreover, *Lactobacillus* was also found to be positively correlated with KOs (K00135, K01956, K01955, and K00265) and metabolites (citric acid and fumaric acid) related to the alanine aspartate and glutamate metabolism pathway; this was also consistent with the results of the previous studies (Ishino et al., 1992; Wang et al., 2010; Kobayashi et al., 2015). Therefore, it was speculated that the cross between domestic and wild boar caused a significant increase in the abundance of *Lactobacillus* in the F4, which significantly increased alanine aspartate and glutamate metabolism and its related KOs, thereby increasing the citric acid and fumaric acid contents. The beneficial gut microbiota and metabolites interacted with each other to play an important role.

However, the reasons leading to the differences of gut microbiota in each group were very complex. The feeding environment and diet composition of pigs in each group were the same, the age was similar, and there was no significant difference in the gut microbiota between pigs of different genders. In recent years, several studies have confirmed that the main transmission mode of gut microbiota was vertical transmission and the important role of host genetic influence on the gut microbiota (Ley et al., 2005; Turnbaugh et al., 2009; Moeller et al., 2018; Raznahan et al., 2018). Therefore, we speculate that hybridization may be the main reason for the differences in gut microbiota among the groups, but more experiments need to be done in the future.

In summary, the composition, function, and metabolites of the gut microbiota of Duroc, F0, F2, and F4 in the wild boar hybrid line were analyzed using multi-omics technologies. The results showed that the gut microbiota of F4 also showed a “dominant” phenomenon similar to the host. The probiotics, beneficial metabolic functions, and beneficial metabolites also significantly increased, and their effects were similar to the dominant characteristics of the host, suggesting that the artificial breeding of animals is important for a healthy gut microbial composition of the offspring. The results provided referential data for the genetics and evolution of gut microbiota as well as microbial-assisted breeding theory.

## Data availability statement

The datasets presented in this study can be found in online repositories. The names of the repository/repositories and accession number(s) can be found in the article/Supplementary material.

## Ethics statement

The animal study was reviewed and approved by the Institutional Animal Care and Use Committee of the Sichuan Agricultural University, Sichuan, China (DKY-2018102014).

## Author contributions

YL: conceptualization, funding acquisition, and supervision. LW: methodology, visualization, and writing—original draft preparation. BZ: software. SZ and FL: resources. SZ and WG: data curation. JZ: validation. LW and FL: formal analysis. WG and JZ: investigation. BZ and YL: project administration, writing—review and editing. All authors have read and agreed to the published version of the manuscript.

## Funding

This work was supported by the Science and Technology Research Program of Chongqing Municipal Education Commission (No. KJQN202202821) and the School-level Project Fund of Chongqing Medical and Pharmaceutical College (No. ygz2022101), and the Sichuan Province Science and Technology Program (No. 2022072), and the project funded by China Postdoctoral Science Foundation (No. 2021M703134), and the Development and Regeneration Key Laboratory of Sichuan Province, Chengdu Medical College (No. SYS20-11), and the China Scholarship Council (No. 202008515065).

## Conflict of interest

The authors declare that the research was conducted in the absence of any commercial or financial relationships that could be construed as a potential conflict of interest.

## Publisher’s note

All claims expressed in this article are solely those of the authors and do not necessarily represent those of their affiliated organizations, or those of the publisher, the editors and the reviewers. Any product that may be evaluated in this article, or claim that may be made by its manufacturer, is not guaranteed or endorsed by the publisher.
