# Supplementary figures and images for: Hybridization altered the gut microbiota of pigs
Source: Front Microbiol. 2023 Jul 3;14:1177947. doi: 10.3389/fmicb.2023.1177947 (PMC10350513; doi:10.3389/fmicb.2023.1177947)

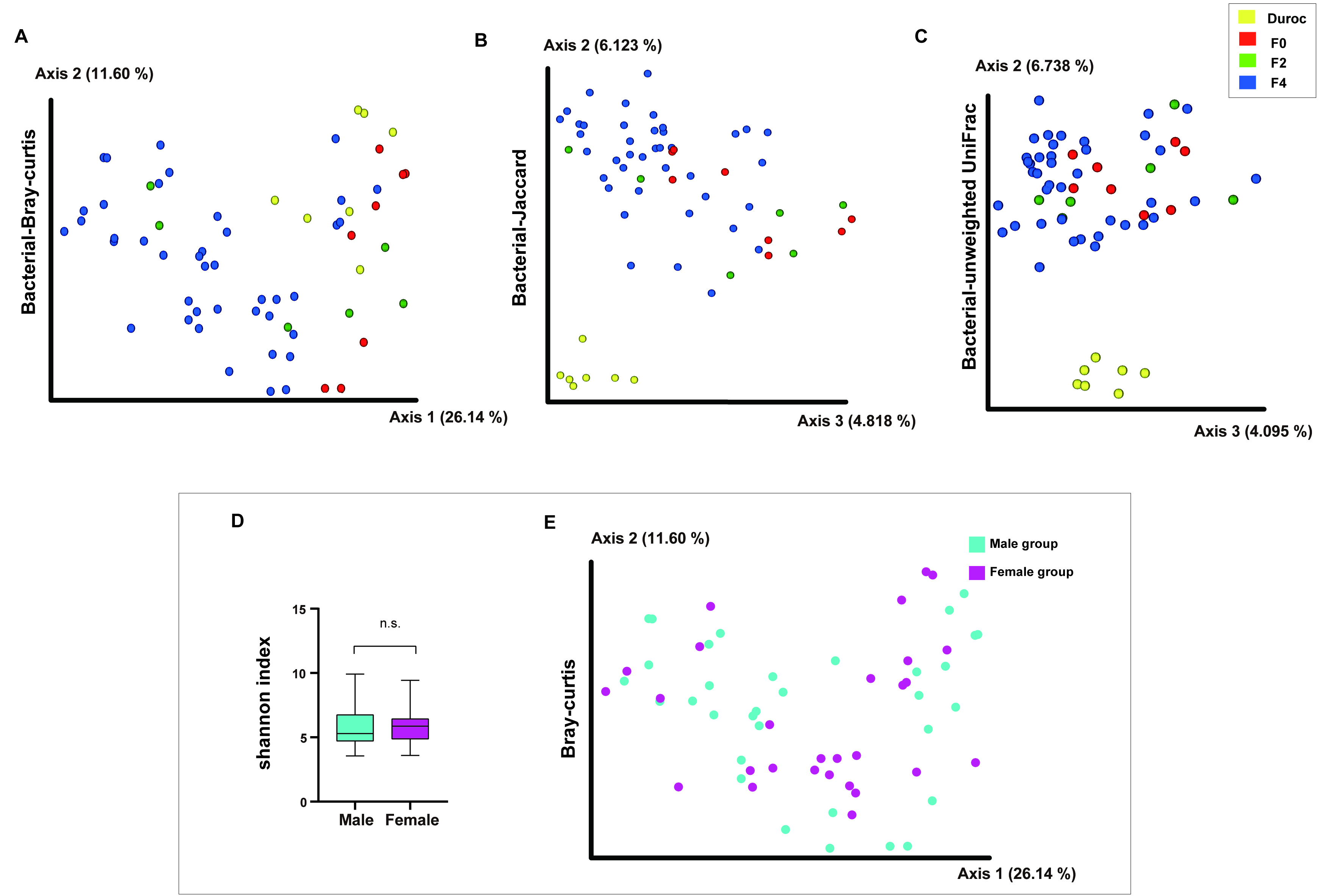

Supplement: Supplementary file 10 [file Image_1.jpg]

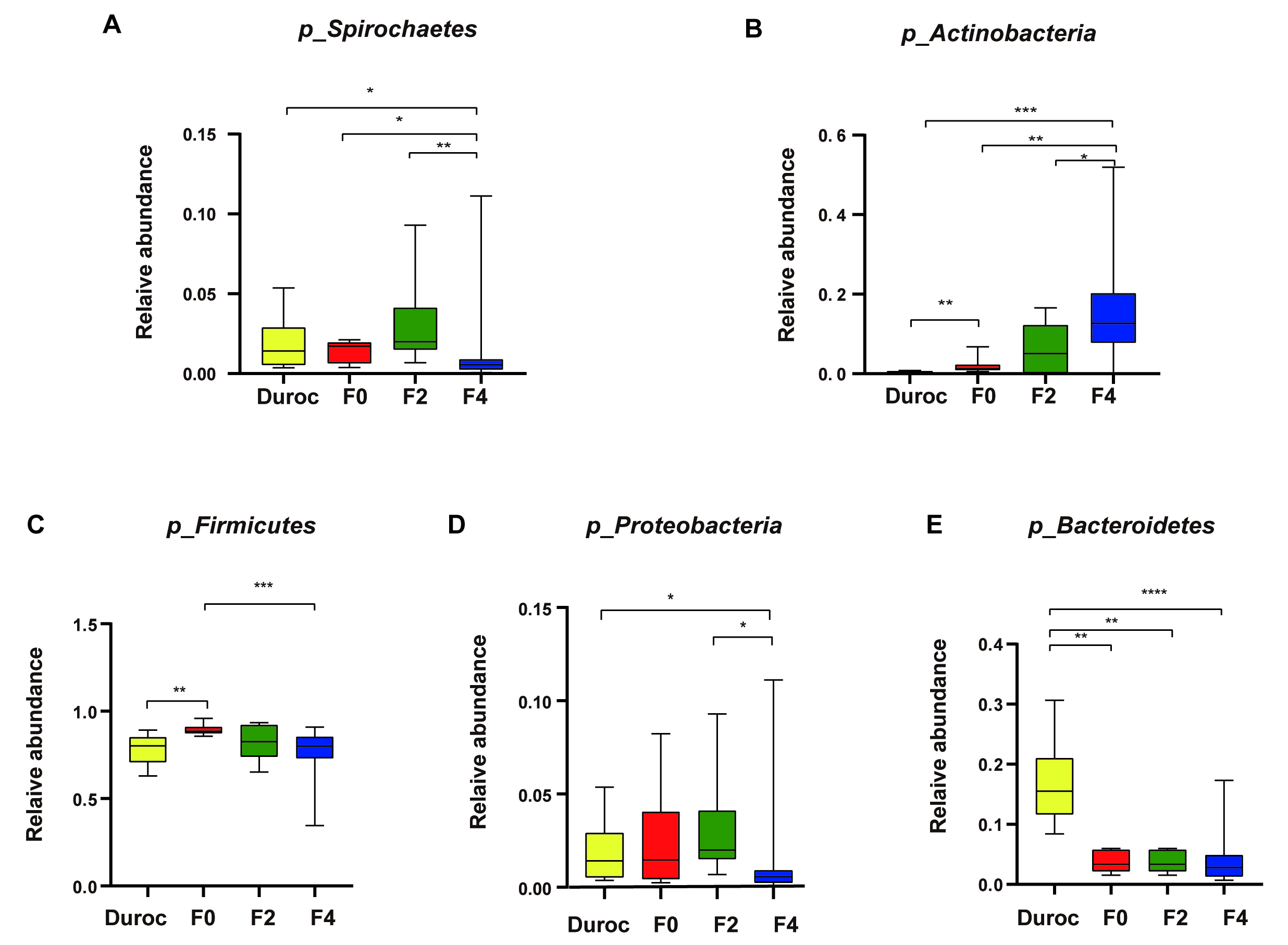

Supplement: Supplementary file 11 [file Image_2.jpg]

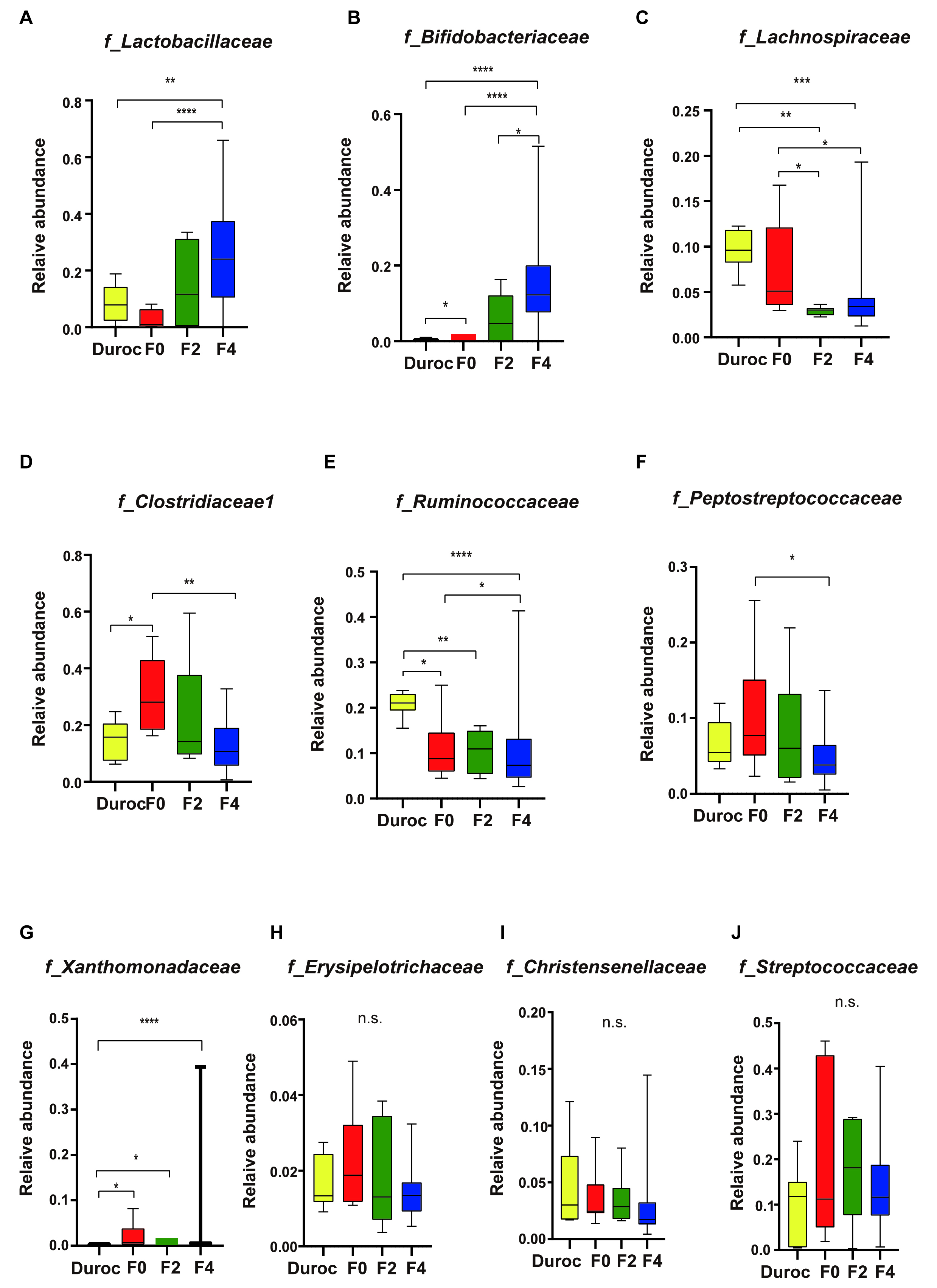

Supplement: Supplementary file 12 [file Image_3.jpg]

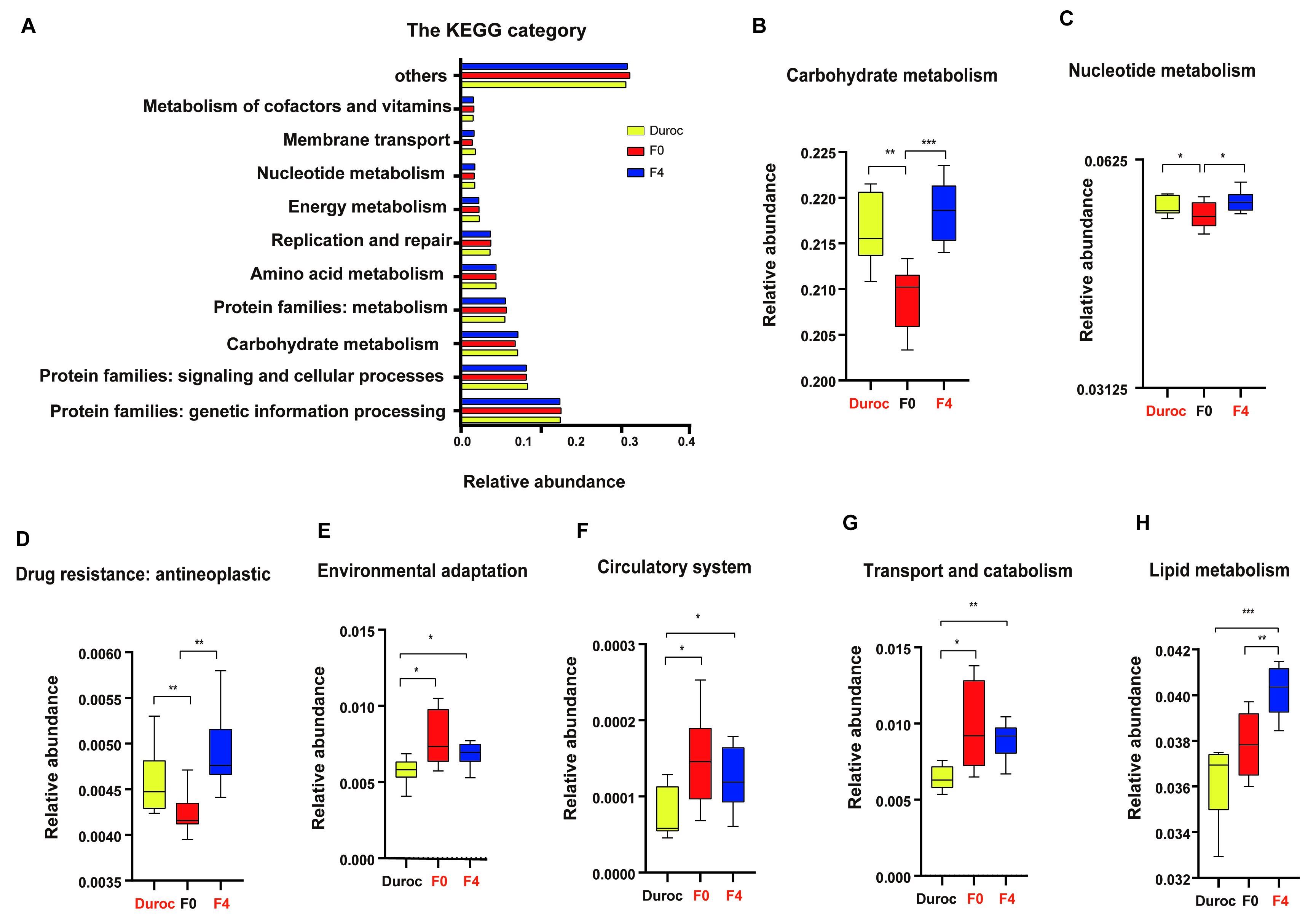

Supplement: Supplementary file 13 [file Image_4.jpg]

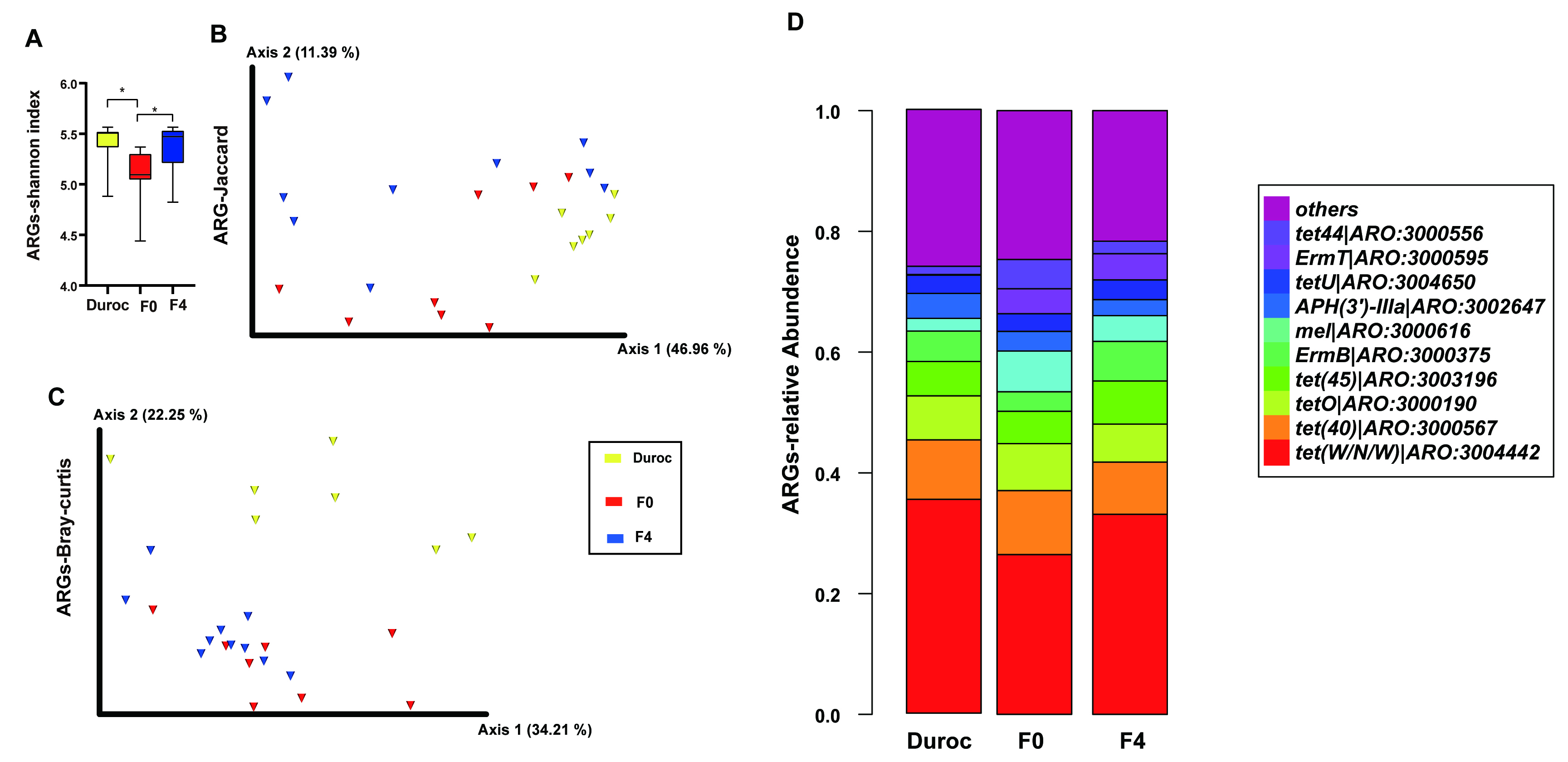

Supplement: Supplementary file 14 [file Image_5.jpg]
